# Tobacco retailer density and smoking behaviour: how are exposure and outcome measures classified? A systematic review

**DOI:** 10.1186/s12889-023-16914-y

**Published:** 2023-10-18

**Authors:** John Baker, Katrin Lenz, Mohd Masood, Muhammad Aziz Rahman, Stephen Begg

**Affiliations:** 1 Department of Community and Allied Health, La Trobe Rural Health School, La Trobe University, Bendigo, Australia; 2 Department of Dentistry and Oral Health, La Trobe Rural Health School, La Trobe University, Bendigo, Australia; 3 Institute of Dentistry, University of Turku, Turku, Finland; 4 School of Health, Federation University, Berwick, Australia; 5 Australian Institute for Primary Care and Ageing, La Trobe University, Melbourne, Australia; 6 Violet Vines Marshman Centre For Rural Health Research, La Trobe Rural Health School, Melbourne, VIC Australia

**Keywords:** Retailer, Density, Smoking, Tobacco, Electronic cigarette, Behaviour, Systematic review, Proximity, Availability

## Abstract

**Introduction:**

To date only a limited number of reviews have focused on how exposure and outcome measures are defined in the existing literature on associations between tobacco retailer density (‘density’) and smoking behaviour (‘smoking’). Therefore this systematic review classified and summarised how both density and smoking variables are operationalised in the existing literature, and provides several methodological recommendations for future density and smoking research.

**Methods:**

Two literature searches between March and April 2018 and April 2022 were conducted across 10 databases. Inclusion and exclusion criteria were developed and keyword database searches were undertaken. Studies were imported into Covidence. Cross-sectional studies that met the inclusion criteria were extracted and a quality assessment was undertaken. Studies were categorised according to the density measure used, and smoking was re-categorised using a modified classification tool.

**Results:**

Large heterogeneity was found in the operationalisation of both measures in the 47 studies included for analysis. Density was most commonly measured directly from geocoded locations using circular buffers at various distances (n = 14). After smoking was reclassified using a smoking classification tool, past-month smoking was the most common smoking type reported (n = 26).

**Conclusions:**

It is recommended that density is measured through length-distance and travel time using the street network and weighted (e.g. by the size of an area), or by using Kernel Density Estimates as these methods provide a more accurate measure of geographical to tobacco and e-cigarette retailer density. The consistent application of a smoking measures classification tool, such as the one developed for this systematic review, would enable better comparisons between studies. Future research should measure exposure and outcome measures in a way that makes them comparable with other studies.

**Implications:**

This systematic review provides a strong case for improving data collection and analysis methodologies in studies assessing tobacco retailer density and smoking behaviour to ensure that both exposure and outcome measures are clearly defined and captured. As large heterogeneity was found in the operationalisation of both density and smoking behaviour measures in the studies included for analysis, there is a need for future studies to capture, measure and classify exposure measures accurately, and to define outcome measures in a manner that makes them comparable with other studies.

**Supplementary Information:**

The online version contains supplementary material available at 10.1186/s12889-023-16914-y.

## Introduction

The distribution of tobacco retailers and the critical influence the tobacco industry has on ensuring its products are ubiquitously available worldwide has been identified as an important component of tobacco control research [1]. The universal availability of tobacco is likely to influence smoking behaviour [2, 3] and exposure to tobacco retailers may influence perceptions about the ease of purchasing cigarettes, the prevalence of smoking, and the personal health consequences of such behaviour amongst young people [4, 5]. It may also normalise the use of tobacco products [6] and encourage tobacco use by providing greater access to tobacco products, marketing (i.e. advertising brand names through price boards), and exposure to other smokers [7]. Greater availability of tobacco products may impact on pricing, with increased competition from retailers possibly lowering cigarette prices [7]. Traditional market theories suggest that the increased availability of consumer goods results in improved consumer awareness, provides greater purchasing opportunities, and contributes to increased sales [8, 9].

The World Health Organization’s Framework Convention on Tobacco Control (WHO FCTC) provides countries with a framework to develop and improve tobacco control legislation and public health prevention and cessation strategies. Internationally, a large number of measures to reduce tobacco supply, accessibility and availability have been implemented. These include the introduction of tobacco retailer licensing systems, restrictions on the types of businesses that can sell tobacco products, and limits to the retail availability of tobacco products, including restrictions on the number of and distance between tobacco retailers within a certain geographical location.

Retail availability of tobacco is typically operationalised in the growing body of research in this area in terms of two related concepts, tobacco retailer density (TRD) and tobacco retailer proximity (TRP). Most studies focus on TRD, which is broadly defined as the number of tobacco retailers within a defined area [10]. Several studies have also examined TRP, which is typically operationalised as the nearest features to a specific origin, such as the most proximal tobacco retailer from a home [11].

A number of systematic reviews [10, 12–15] and meta-analyses [2, 16] have attempted to assess associations between TRD and smoking behaviour in studies focusing on both youth and adults. These reviews have documented statistically significant associations, particularly TRD around participants’ homes or activity spaces and smoking or e-cigarette behaviours.

A narrative review of nine studies on TRD and TRP and adolescent smoking only amongst licensed tobacco retailers in North America found associations between those factors and lifetime smoking (two studies), past 12-month smoking (one study), past 30-day smoking (eight studies), as well as susceptibility to smoking (two studies) [10]. A meta-analysis of 11 studies on the relationship between TRD and adolescent smoking found significantly higher rates of smoking with greater density around homes but not schools, however this study only included one smoking outcome measure (past-month smoking) and only focused on youth [2]. A systematic methodological review of 20 studies on the associations between TRD, TRP and smoking amongst young people aged 12–25 years, found positive associations in two studies and a negative association in one study in the four studies identified as having high methodological quality [12].

Since this systematic review commenced, two other systematic reviews have been published that discuss the results of studies looking at TRD and TRP and smoking behaviour. One of those systematic reviews [13] summarised associations between TRD and TRP to homes, schools and communities, and smoking behaviours across 35 studies focusing on those aged 18 years and younger. It found that the existing literature supported a positive association between TRD and smoking behaviours near youths’ homes, regardless of the density measure used, while one study included for review found an association between TRD around activity spaces and smoking behaviour, but associations were not found between TRP and smoking behaviour.

The second systematic review assessed geographic measures of TRD and TRP and smoking behaviour across 40 studies [14]. It found nearly half of TRD studies measured retailer counts within an area, while more than 80% of studies included in this systematic review measured TRP did so through measuring length distances using the street network. Greater TRD was generally associated with higher smoking prevalence, increased smoking initiation, and lower cessation outcomes. TRP measures were only associated with cessation outcomes, with closer proximity to retailers associated with reduced cessation rates and quitting outcomes amongst current smokers.

Substantial differences in the way both exposure and outcome measures are defined have made it difficult to compare and meta-analyse the existing TRD literature [12]. The review by Marsh et al. [13] stated that inconsistent associations between TRD and smoking behaviour may be as a result of the outcome variables used, and highlighted various ways that outcome measures are classified and defined, but did not investigate this further. To date, several meta-analyses [2, 16] have been published focusing on associations between TRD and smoking behaviour. One of those studies [2] noted that inconsistencies in results may be as a consequence of study factors, including smoking outcomes, and that there was a lack of consistency in how past-month smoking behaviour was defined in the literature. The other [16] concluded that regardless of how TRD or TRP are measured or what country the research was conducted in, reducing the density and proximity of tobacco retailers is consistently associated with reductions in adult tobacco use. However, while this analysis considered a broad range of smoking-related behaviours (e.g. initiation; smoking; quitting; relapse and psychological constructs directly related to quitting, such as smoking urges, pro-cessation attitudes and self-efficacy), it did not discuss how smoking was measured in the 10 studies that were included in the sub analysis for this outcome.

Although it is important to ensure both exposure and outcome measures are clearly defined and captured in the literature, no reviews to date have focused on examining how exposure measures (i.e. TRD) and outcome measures (smoking behaviour) are defined and classified, and how this might affect the precision of results.

The current review sought to address this gap by examining the existing evidence on associations between TRD and smoking behaviour from the perspective of summarising how these variables have been measured in the literature. Studies that assess retail tobacco availability predominantly analyse associations between TRD and smoking behaviour or electronic cigarette use (e-cigarette), therefore these studies were the main focus for this review.

A descriptive approach was adopted with the aim of addressing the following questions: (1) what approaches to measure TRD and smoking behaviour have researchers adopted in this field of research? and (2) what gaps in the evidence base have developed as a result of these approaches?

## Methods

### Literature search strategy

The systematic review was registered with PROSPERO (no. CRD42017082385). Minor changes to the aims of the study but not the search strategy were recorded in PROSPERO after the review commenced in response to two new published reviews that had similar aims. PRISMA-P guidelines were used to assist the review process. Search terms using keyword and text searches only included ‘Outlet density AND smoking’, ‘Retail density AND smoking’, ‘Smoking AND convenience store’, ‘Smoking OR tobacco AND density’, ‘Tobacco outlet density AND smoking’, and ‘Tobacco retailer density AND smoking’ (Supplementary Table 1).

CINAHL, Cochrane, Medline, ProQuest, PsycArticles, PsycINFO, PubMed, Scopus, SocINDEX, and Web of Science databases were searched. Two literature searches were conducted. The first between March and April 2018 and the second in April 2022. Searches were not limited by country, language, date or peer-review status.

**Fig. 1:**
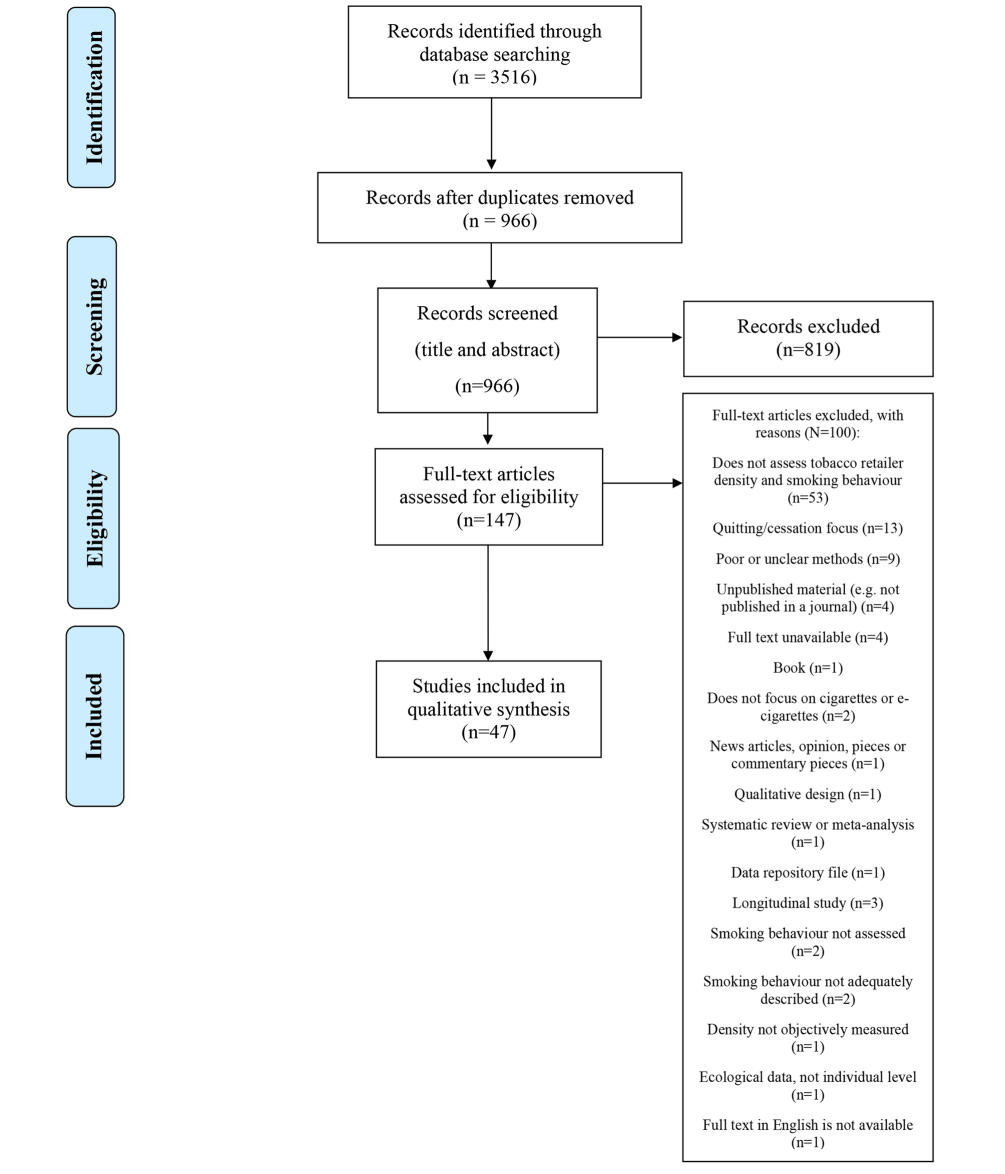
Tobacco retailer density and smoking behaviour: Systematic Review flow chart

All studies identified in database searches were imported into Covidence software and duplicate studies were identified and removed (Fig. 1) [17]. Titles and abstracts were screened independently by two researchers (SB & MM first search, JB & KL second search) and conflicts were resolved by a third researcher (MAR first search, SB second search). The included full text articles were divided into two sections and reviewed by author pairs (SB & JB reviewed 61 articles and MM & MAR reviewed 60 articles in their initial search. JB & KL reviewed 13 articles and MM & MAR reviewed 13 articles in their second search). Conflicts were sent to a representative of the other team (JB & MM first search, JB second search) or to another researcher (SB second search) for final review. The reasons for exclusion were recorded in Covidence. The researchers were not blinded to the titles, study authors or institutions.

### Inclusion and exclusion criteria

Cross-sectional studies examining TRD and the experimentation, uptake and continuation of smoking behaviour were included for review. This review only included cross-sectional studies that assessed associations between the availability of tobacco and e-cigarette products and smoking or e-cigarette use experimentation, uptake and/or continuation, as there is a large body of evidence examining associations between these variables. There were only a small number of studies that assessed tobacco and e-cigarette retailer density and cessation, and these were typically longitudinal in nature. Therefore, studies that looked at cessation outcomes were excluded as tobacco and e-cigarette availability and effects on experimentation and uptake were deemed to be the most important factor in leading to contributing to tobacco or e-cigarette use, particularly amongst youth and young people.

This study focused on cigarettes and e-cigarette use as these are typically the most common tobacco and nicotine-containing products sold. Therefore, smoking behaviour was defined as cigarette and e-cigarette use, but not other forms of tobacco use (e.g. smokeless tobacco, chewing tobacco, snus, dip, shisha, hookah etc.).

Cigarettes were included in this definition as cigarette consumption is increasing in many countries (particularly in low- and middle-income countries), cigarettes are popular amongst youth, and smoking-related questions commonly focus on the use of these products [18, 19]. E-cigarettes were included because the use of these products is an emerging public health concern across many countries and these devices may lead to cigarette use amongst youth [20–23].

Studies were excluded if they did not provide details on the study population(s), a definition of how TRD was measured (e.g. whether density was measured through circular buffers at set distances), or a definition of smoking behaviour. Studies were also excluded if smoking behaviour was only estimated, broadly captured or not captured at all. For example, studies by Chaiton et al. [24] and Pearce et al. [25] did not provide details on the type(s) of smoking behaviour being measured (e.g. current or past-month smoking). Other exclusion criteria were: systematic reviews or meta-analyses, studies where a full-text article was unavailable or not available in English, studies with poor or unclear methods, unpublished studies (e.g. not published in a peer-reviewed journal), books, news articles, opinions, pieces or commentary pieces, studies with a qualitative design, studies with ecological data, and longitudinal studies. Longitudinal studies were excluded because they largely focused only on cessation and relapse rather than experimentation, uptake and continuation.

### Data extraction

Extracted data included the Covidence article reference number, the title, study authors, year of publication, the TRD measure used and the smoking behaviour definition(s) used. Data relating to how studies identified tobacco and e-cigarette retailers (e.g. through validating tobacco existing tobacco and e-cigarette retailer lists) was not extracted. The setting for the study was another attribute of interest. This included participants’ home, school, or activity space for example. Activity spaces are defined as all of the locations an individual personally experiences as a result of their daily activities, [26] and consist of the locations and the routes that a person has travelled to or visited [27].

Studies that measured distances in miles were converted into kilometres (km). All extracted information from each included study was extracted independently by two researchers (JB & SB first search, JB & KL second search) using an Excel template. The two researchers (JB & SB, JB & KL) then validated the extracted data through discussion to ensure accuracy and consistency, and any discrepancies were resolved.

### Quality assessment

The quality of the studies was assessed using the National Heart, Lung and Blood Institute’s (NHLBI) Quality Assessment Tool for Observational Cohort and Cross-Sectional Studies [28]. During the first search the studies were divided into two sections and the quality of each study was classified by author pairs: 18 articles by SB & JB and 17 articles by MM & MAR (Supplementary Table 2). Disagreements on the overall quality rating (Good/Fair/Poor) were resolved through discussion, and if agreement could not be made then conflicts were sent to a representative of the other team (JB & MM) for a final decision. In the second search, all 12 studies were classified by JB & KL (Supplementary Table 2) and if agreement could not be made then conflicts were sent to SB. Any discrepancies were resolved by reaching consensus.

### Smoking behaviour measures

For this review, the term smoking behaviour included both cigarette and e-cigarette use. The smoking of cigarettes and e-cigarettes is ideally assessed with respect to at least the following dimensions: recency, frequency and intensity. However, the questions asked in most of the studies reviewed in the screening phase of this review tend to focus on only one of these dimensions (typically the first), or two at most. To assist with classifying the studies during the synthesis phase, the research team developed a smoking behaviour classification tool based on work by Mayhew, Flay & Mott [29]. For some studies, the labels attached to a certain smoking behaviour had a different meaning when considered in the light of this classification tool. Where possible the underlying questions asked of participants were used to determine the most appropriate category to apply, not the labels. To ensure consistency, two members of the research team (SB & MM) each reviewed 12 (approximately 33% each or 66% of total) randomly selected articles to apply the smoking behaviour categories. These were then compared to the smoking behaviour categories applied by the primary researcher (JB) and any discrepancies were discussed and resolved. Studies that measured only one type of smoking behaviour were grouped with other studies that measured only that behaviour. Studies that measured more than one behaviour were grouped with other studies that measured the same combination of behaviours. The grouping of studies in this way allowed for many combinations of behaviour categories.

## Results

In total, 3,516 articles were identified through database searches and imported into Covidence (Fig. 1) [17]. Following removal of duplicates, 966 unique articles were identified of which 147 articles remained after initial screening of titles and abstracts. Full text review resulted in the exclusion of 100 studies for specific reasons and a final sample of 47 studies for qualitative synthesis.

### Overview of studies

The 47 studies included in this systematic review were published between 2005 and 2022 (Supplementary Table 2). Thirty-three studies were assessed as ‘Good quality’, whilst 14 studies were rated as ‘Fair quality’. The studies were undertaken in the United States (n = 29), Canada (n = 7), Australia (n = 4), the United Kingdom (n = 3), New Zealand (n = 2), India (n = 1) and South Korea (n = 1). The smallest participant sample size was 100 [30] whilst the largest sample size was 88,850 [31]. Twenty-seven studies collected data from specific age groups (e.g. 13–18 years) and 13 of the 47 studies collected data from school students (e.g. middle and high school grades). One study did not report sample characteristics [32] and six studies stated that ‘Adults’ or ‘Young Adults’ participated, without providing an age range.

### Smoking behaviour measures

Several differences between the Mayhew et al. smoking classification approach for youth and the one ultimately adopted in this review are worth noting (Table 1). First, since the focus of this review was on existing smoking behaviour, Mayhew et al.’s ‘Non-smoking-contemplation and preparation stages’ category was removed and a ‘Quit/Stopped/Ex-smoker’ category was added to accommodate studies in which participants were asked whether they have previously smoked but had not smoked on any day in the past month. Second, the smoking category ‘Tried’ was merged into ‘Ever-tried’ and the one-year timeframe was removed because most studies did not place limits on this aspect of smoking behaviour. Third, the ‘Experimenter’ stage was removed because the definition of ‘Smokes occasionally on an experimental basis’ and the associated measurements were quite prescriptive and did not fit with the broader smoking behaviours being assessed in the studies. Fourth, the ‘Regular’ and ‘Established/daily smoker’ stages were subdivided by recency into the following categories: ‘Past-year smoking’, ‘Past-month smoking’, and ‘Current smoking’. For the past-year and past-month categories, the intensity threshold was reworded from ‘Smoked more than once’ to ‘Smoked at least one cigarette.’ ‘Current smoking’ was defined as ‘any current smoking behaviour or any smoking in the past week’. And lastly, reference to total lifetime cigarette consumption (+/- 100 cigarettes) was removed because most of the studies did not assess this.

**Table 1:** Smoking measures classification tool for youth adapted from Mayhew, Flay, & Mott

Measure	Intensity	Recency	Frequency
**Ever-tried**	Tried a puff or two or smoked one or two cigarettes	Any time	Tried a puff or two or smoked one or two cigarettes then stopped OR can be categorised to one of the stages below if question asked about further smoking
**Current smoking**	Indicates any current smoking behaviour	Present time or past week use	Daily/nearly daily/some days/occasionally
**Past-month smoking**	Smoked at least one cigarette in past month	Past month (30 days)	Daily/nearly daily/some days/occasionally within past month
**Past-year smoking**	Smoked at least one cigarette in past year	Any use in past year	Daily/nearly daily/some days/occasionally within past year
**Quit/Stopped/** **Ex-smoker**	Has not smoked a cigarette in past month	No use in past month	Previously classified as a current, past-year or past-month smoker but has not smoked on any day in past month

### Smoking measures extracted

Smoking behaviour measures varied widely across studies, and numerous terms were used in the literature to describe smoking behaviour (Supplementary Table 3). Once smoking behaviours were reclassified, *ever-tried smoking* was captured in 11 studies, while 26 studies measured *past-month smoking*, 20 studies measured *current smoking* and 4 studies measured *past-year smoking*. Some studies included more than one smoking measure, such that the permutations of these categories were as follows: *ever-tried* and *past-month* (n = 5), *ever-tried* only (n = 1), *past-month* and *current* (n = 1), *past-month* only (n = 17), *current* only (n = 15), *past-year* only (n = 2), *past-year* and *past-month* (n = 1), *ever-tried*, *past-year* and *past-month* (n = 1), *ever-tried* and *current* (n = 3) and *ever-tried*, *past-month* and *current* (n = 1).

There was much consistency in the definition of *ever-tried* smoking across studies, with most studies describing this type of smoking as ‘One or more times’ or ‘Ever tried/used’. A number of studies used definitions for different smoking behaviours that did not appear to be overtly distinct from one another. For example, McCarthy et al. [33] defined both ‘Established smoking’ and ‘Experimental smoking’ as ‘Smoking at least one cigarette in the past 30 days’, however only those who indicated smoking more than 100 cigarettes in their lifetime were classified as ‘Established smokers’. Both of these smoking behaviours were classified as *past-month smoking* as this better reflected the time period that respondents were asked to recall smoking behaviour.

### Tobacco retailer density measurements

Of the 47 studies included, TRD was classified into two broad groups: those that measured density directly from specific geocoded locations, and those that utilised Kernel Density Estimates (KDE) (Supplementary Table 4). KDE is used to create a continuous density surface of the intensity of exposure that takes into account the number of tobacco retailers within the kernel, and weighting them by their proximity from the point of measurement (e.g. from homes or schools) [11, 34].

Fourteen studies assessed TRD using circular buffers from geocoded locations (e.g. home or school), with radii ranging from 0.4 km to 1.6 km in size. The most common measure was a 0.8 km (1/2 a mile) radii from schools (n = 5). The majority of studies analysed TRD directly from geocoded locations, including schools (n = 11), homes (n = 2), or both (n = 1).

Nine studies directly assessed TRD through generating network service areas represented as polygons or polylines around geocoded locations, including homes, schools and activity spaces using the street network or footpaths to measure a specified distance. These distances ranged from 0.1 km to 1.6 km. For one of the studies, TRD within activity spaces was defined as the mean number of, or proximity to, tobacco retailers across regular activity locations (e.g. when studying, working, grocery shopping, undertaking physical activity, leisure activity and two other activities) selected by respondents [35]. Another study measured density by the number of tobacco shops within 100 m of polylines of activity spaces on each day, where activity spaces referred to places people frequent as part of their daily routine (e.g. parks, shopping centres, city centres and schools) [30].

Ten studies assessed direct TRD per population (e.g. number of retailers per 1,000 people) using neighbourhoods, census tracts, cities or counties as the defined geographical area. Five studies assessed direct TRD through the number of tobacco retailers per-km of roadway (e.g. per-1.6 km to per-50 km).

Four studies measured the number of retailers per population using Kernel Density Estimates (KDE), while four other studies measured the number of retailers per-km^2^ using KDE, and one study measured the number of retailers per postcode using KDE.

Two studies assessed TRD using the number of retailers per-km^2^ using suburbs or census tracts as the defined geographical areas. In the context of this research, suburbs are officially gazetted boundaries in cities and larger towns, and localities elsewhere [36].

## Discussion

This systematic review described the methods used in the existing literature to capture and measure TRD and smoking behaviour. The methods for measuring TRD varied across the literature, however the most common approach assessed density directly through applying circular buffers at varying distances from specific geocoded locations, such as homes or schools, using GIS software (n = 14). Smoking behaviour was also described in many ways, however once reclassified using the available information, past-month smoking behaviour was most frequently captured (n = 17).

### Smoking behaviour

A plethora of outcome measures are used to describe smoking behaviour by international agencies including the WHO and national bodies such as Ministries of Health. The studies included in this systematic review also used a wide range of smoking descriptors, such as experimentation, occasional, non-daily, regular, and daily smoking. For example, several studies described ‘Current smoking’ as ‘Any smoking in the past 30 days’, while other studies described it as ‘Currently smoking daily or occasionally’, with the former considered to reflect *past-month smoking* and the latter *current smoking* once re-classified by the researchers in the current review. It was therefore evident in the literature that smoking behaviour measures were complex and often inconsistent, [29] making them difficult to interpret.

The most common way that studies measured smoking behaviour was *past-month smoking*. Self-reported smoking behaviour was the most common data collection method used in studies, but this may under-report true smoking behaviour due to potential social desirability bias [37] or recall decay, particularly if respondents are asked to remember their smoking behaviour over a long period of time (e.g. on how many days in the past 30 days respondents smoked and the average number of cigarettes smoked on those days).

A consistent approach to capturing and classifying different smoking behaviours is important so that comparisons across different studies can be undertaken. One reason for methodological inconsistencies in capturing smoking behaviour is the absence of clear guidance on best-practice approaches when exploring outcome measures. Although previous research on TRD and smoking behaviour has acknowledged these inconsistencies, possible solutions are limited. In 2011, the Global Adult Tobacco Survey Collaborative Group developed a guide for surveys that include questions focusing on tobacco use, [38] however many of the survey instruments used to collect data in the studies appear not to have followed its recommendations. More widespread usage of this resource would strengthen future research into TRD and smoking behaviour.

This analysis identified subtle differences in wording across various types of smoking behaviour within studies. For example the study by Chan et al. [39] defined daily smoking as ‘smoking every day or almost every day in the past 30 days’ and defined occasional smoking as ‘smoking some days or only 1 or 2 days in the past 30 days’. Both smoking behaviours were reclassified as *past-month smoking* according to the classification tool developed for the present analysis since it focuses on the recency of the smoking behaviour, not its intensity. A strength of this tool, therefore, is that it provides an objective basis for summarising and sorting the most commonly used questions about smoking behaviour. Its application would facilitate comparison between TRD and different smoking behaviours in future systematic reviews and meta-analyses.

### Tobacco retailer density

Most studies focused on assessing TRD and smoking behaviour from participants’ homes or schools primarily using circular buffers at varying distances. Reasons for choosing a particular buffer size included practical constraints, [40] the buffer distance was approximately a 10 minute walk from school, [41] or the distance was assumed to be the outer limit that most students would walk or cycle regularly to school in urban settings, and the minimum distance necessary to encompass at least one tobacco retailer amongst students who attend a rural school [33]. Although the use of circular buffers is a simple and reliable method for measuring TRD, studies using this approach did not take into account the built environment, which could increase the true travel time or distance to retailers. For example, a participants’ home or school could be geographically close to several tobacco retailers using circular buffers; however buildings, fences, highways, waterways or other physical features might prevent direct travel to those retailers, thus increasing the true distance and/or travel time. It is possible that earlier studies may have resorted to using circular buffers to measure TRD as GIS software did not provide access to KDE measures. GIS software has evolved over time, giving researchers more nuanced measures of exposure to retailer presence in the community than was available historically.

Therefore studies that measured density using network service areas (e.g. the street network or footpaths) represented as polygons or polylines around geocoded locations appear to be a more valid and reliable measure of TRD, as they take the physical environment into account. Valiente et al. [14] recommend that TRD is measured through both length-distance and travel time using the street network and footpaths, and weighted by the size of an area, population, or road length, or measured using KDE. [14].

The Uncertain Geographic Context Problem also represents an inherent limitation in the definition of exposure measures used to analyse the real influence of the environment on population health, and few studies captured data on the time and duration that participants spent within defined areas (e.g. homes or schools) and/or the length of time spent exposed to tobacco retailers whilst in these areas [42, 43]. Studies focusing beyond participants’ home and school environments would likely add value to this field of research and provide a better understanding of how regular interactions with tobacco retailers in the broader environment (i.e. activity spaces) may influence smoking behaviours [44].

Few studies included in this systematic review assessed possible associations between TRD within daily activity spaces and smoking behaviour. Studies focusing only on home or school environments may not take into account adolescents’ increased autonomy, mobility and social networks that extend beyond these two settings [45, 46]. Only two studies in the current systematic review analysed TRD and smoking around activity spaces [30, 35]. The inability to collect data on respondents’ movements across activity spaces may be due to the complex technical nature of tracking participants over an extended period of time and over geographically large areas [42]. However smartphone technologies allow real-time data to be easily captured from participants, providing much more detailed information on individuals’ interactions with tobacco retailers in the broader environment during day-to-day activities, instead of relying on self-report data [42, 47]. Research using Ecological Momentary Assessment in defined populations (e.g. adolescent smokers) to identify where and when they make retail tobacco purchases may be useful. The literature assumes that TRD and TRP are major predictors of where adolescents purchase tobacco products, however a more important criterion may be a retail source not likely to be observed by parents or neighbours for example. As better GIS software and GPS-enable devices become available, more nuanced investigations may be able to explore observed associations between a community’s tobacco retailers and tobacco use within that community.

The included literature also highlighted differences in measuring TRD across varying geographical areas, such as urban, regional and rural districts [32]. It has been suggested [48] that TRD measures in non-metropolitan areas might need to be adjusted to account for lower population densities and greater travel distances to tobacco retailers. For example, McCarthy et al. [33] explicitly justified their buffer in part to facilitate capturing rural students’ exposures to rural tobacco retailers. Although this systematic review did not compare studies by metropolitan or non-metropolitan locations, most studies focused on metropolitan areas, with only four studies [32, 33, 49, 50] analysing associations between TRD and smoking behaviour in non-metropolitan settings. Existing research suggests that TRD may be greater in non-metropolitan areas, [51, 52] therefore it is important for future research to explore associations between TRD and smoking behaviour in these areas [50].

Previous research has also identified mixed sensitivity when auditing existing tobacco retailer databases, such as those generated through licensing or registration systems or through commercial sources [53–55]. For large geographical areas such as major cities, it is recommended that researchers conduct random sample ground-truthing to establish the proportion of tobacco retailers likely to have been overcounted (e.g. no longer selling tobacco) or undercounted (e.g. new businesses that have not yet registered) to accurately describe how well a database represents the population of interest, based on just a small random sample of the areas being studied.

For research in smaller geographical areas, future studies could attempt to verify all tobacco retailers in existing databases and to identify other tobacco retailers operating through field visits within defined geographical areas prior to collecting data on smoking behaviour [55]. This may increase the accuracy of TRD and provide a more precise representation of the true exposure to tobacco retailers amongst participants.

The current systematic review did not include longitudinal studies. Longitudinal studies are important to determine whether causal relationships exist between TRD, smoking behaviour and cessation amongst both youth and adults, and to identify protective factors that might reduce or prevent associations between tobacco availability and smoking behaviour. Currently it is unclear whether TRD has a particular effect on certain types of smoking behaviour, however longitudinal or cohort studies could examine these relationships over time. A recent systematic review [13] included one longitudinal study and found a positive association between TRD and smoking. It should be noted that although longitudinal or cohort studies are superior to cross-sectional studies in identifying causal direction, they are subject to similar model misspecification due to the uncontrolled, unmeasured confounders that may explain observed associations. Nuyts et al. [12] highlights the importance of quasi-experimental studies, which take advantage of natural experiments when, for example, jurisdictions adopt tobacco retailer density and proximity restrictions near schools.

We agree with Marsh et al. that inconsistencies in how the exposure and outcome measures are defined are likely to play a role in the reported associations between TRD and smoking behaviour. The results from existing systematic reviews and meta-analyses should therefore be interpreted with caution, and future systematic reviews would be strengthened by a more consistent approach to measuring TRD and smoking behaviour in the literature. We note, however, that as more editors and funders demand that authors place the data on which their work is based in publicly-accessible repositories, it will become increasingly possible to overcome at least some of these inconsistencies by undertaking secondary analysis of the publicly available data.

Studies were not grouped by country or location and were not categorised according to existing tobacco retail policy approaches, such as minimum pricing legislation, PoS display bans, tobacco advertising bans, tobacco retailer licensing systems or minimum-distance laws. This systematic review did not compare studies that focused on the sale of e-cigarettes only and its association with uptake. However, future research could certainly look at this, particularly given the significant uptake of e-cigarette use amongst youth globally.

Jurisdictions have taken different approaches to retail tobacco and e-cigarette sales legislation and these factors may play important roles in the promotion and normalisation of tobacco and e-cigarette products in the community (through product advertising, for example) [40].

It is also important to recognise that much of the existing literature tends to focus on determining whether density (and/or TRP) contributes to smoking behaviour, however it would be appropriate for future research to determine whether certain policies that address density (and/or proximity) may prevent smoking behaviour and/or improve smoking cessation outcomes for existing smokers who are attempting to quit. Retailer licensing systems can be utilised to reduce access to and availability of tobacco and e-cigarette products, while providing important data for research on the availability of tobacco and e-cigarette products and their associations with experimentation, uptake, continuation and cessation.

## Conclusions

In conclusion, TRD and smoking behaviour were defined and measured in the existing literature using different terms and descriptors. After classification, measuring TRD using circular buffers at varying distances was the most common approach. It is recommended that TRD is measured through length-distance (i.e. generating polygons) and travel time using the street network and footpaths, and weighted by the size of an area, population, road length, or using KDE. Future research should also focus on measuring exposure to tobacco retailers in broader activity spaces beyond homes and schools to gain a better understanding of associations between TRD and smoking behaviour throughout daily life. After reclassification, past-month smoking was the most common smoking type measured in the literature. The consistent application of a smoking measures classification tool, such as the one developed for this systematic review, would enable more robust comparisons between studies that assess TRD and smoking behaviour. The findings from this systematic review also support the introduction of comprehensive tobacco and/or e-cigarette retailer licensing systems to provide precise data on levels of exposure (i.e. tobacco and e-cigarette retailers) and smoking and/or e-cigarette behaviour outcomes. The findings highlight the need for future studies to capture, measure and classify both exposure and outcome measures accurately and consistently to enable better comparisons with other studies and other jurisdictions.

### Electronic supplementary material

Below is the link to the electronic supplementary material.

Supplementary Material 1

Supplementary Material 2

Supplementary Material 3

Supplementary Material 4

## Data Availability

The data underlying this article will be shared on reasonable request to the corresponding author.
