# Supplementary material for: Tobacco retailer density and smoking behaviour: how are exposure and outcome measures classified? A systematic review
Source: BMC Public Health. 2023 Oct 18;23:2038. doi: 10.1186/s12889-023-16914-y (PMC10585801; doi:10.1186/s12889-023-16914-y)
Supplement: Supplementary file 1 — Supplementary Material 1 [file 12889_2023_16914_MOESM1_ESM.docx]

Supplementary Table 1.

Search strategy and results: CINAHL (EBSCO)

| **#** | **Searches** | **Results** | **Search Type** |
| --- | --- | --- | --- |
| 1 | Outlet density AND smoking | 36 | Standard |
| 2 | Retail density AND smoking | 17 | Standard |
| 3 | Smoking AND convenience store | 74 | Standard |
| 4 | Smoking OR tobacco AND density | 0 | Standard |
| 5 | Tobacco outlet density AND smoking | 29 | Standard |
| 6 | Tobacco retailer density AND smoking | 24 | Standard |

Search strategy and results: Cochrane

| **#** | **Searches** | **Results** | **Search Type** |
| --- | --- | --- | --- |
| 1 | Outlet density AND smoking | 5 | Standard |
| 2 | Retail density AND smoking | 6 | Standard |
| 3 | Smoking AND convenience store | 26 | Standard |
| 4 | Smoking OR tobacco AND density | 274 | Standard |
| 5 | Tobacco outlet density AND smoking | 5 | Standard |
| 6 | Tobacco retailer density AND smoking | 4 | Standard |

Search strategy and results: Medline (Ovid)

| **#** | **Searches** | **Results** | **Search Type** |
| --- | --- | --- | --- |
| 1 | Tobacco retailer density AND smoking | 25 | Standard |
| 2 | Outlet density AND smoking | 53 | Standard |
| 3 | Retail density AND smoking | 15 | Standard |
| 4 | Smoking AND convenience store | 37 | Standard |
| 5 | Smoking OR tobacco AND density | 0 | Standard |
| 6 | Tobacco outlet density AND smoking | 36 | Standard |

Search strategy and results: ProQuest

| **#** | **Searches** | **Results** | **Search Type** |
| --- | --- | --- | --- |
| 1 | Outlet density AND smoking | 16 | Standard |
| 2 | Retail density AND smoking | 22 | Standard |
| 3 | Smoking AND convenience store | 71 | Standard |
| 4 | Tobacco outlet density AND smoking | 15 | Standard |
| 5 | Tobacco retailer density AND smoking | 15 | Standard |
| 6 | Smoking OR tobacco AND density | 0 | Standard |

Search strategy and results: PsycINFO (Ovid)

| **#** | **Searches** | **Results** | **Search Type** |
| --- | --- | --- | --- |
| 1 | Outlet density AND smoking | 197 | Standard |
| 2 | Retail density AND smoking | 18 | Standard |
| 3 | Smoking AND convenience store | 89 | Standard |
| 4 | Tobacco outlet density AND smoking | 94 | Standard |
| 5 | Tobacco retailer density AND smoking | 63 | Standard |
| 6 | Smoking OR tobacco AND density | 0 | Standard |

Search strategy and results: PsycArticles (Ovid)

| **#** | **Searches** | **Results** | **Search Type** |
| --- | --- | --- | --- |
| 1 | Outlet density AND smoking | 7 | Standard |
| 2 | Retail density AND smoking | 1 | Standard |
| 3 | Smoking AND convenience store | 14 | Standard |
| 4 | Tobacco outlet density AND smoking | 3 | Standard |
| 5 | Tobacco retailer density AND smoking | 4 | Standard |
| 6 | Smoking OR tobacco AND density | 0 | Standard |

Search strategy and results: PubMed

| **#** | **Searches** | **Results** | **Search Type** |
| --- | --- | --- | --- |
| 1 | Outlet density AND smoking | 76 | Standard |
| 2 | Retail density AND smoking | 108 | Standard |
| 3 | Smoking AND convenience store | 102 | Standard |
| 4 | Tobacco outlet density AND smoking | 69 | Standard |
| 5 | Tobacco retailer density AND smoking | 80 | Standard |
| 6 | Smoking OR tobacco AND density | 0 | Standard |

Search strategy and results: Scopus

| **#** | **Searches** | **Results** | **Search Type** |
| --- | --- | --- | --- |
| 1 | Outlet density AND smoking | 101 | Standard |
| 2 | Retail density AND smoking | 92 | Standard |
| 3 | Smoking AND convenience store | 152 | Standard |
| 4 | Tobacco outlet density AND smoking | 85 | Standard |
| 5 | Tobacco retailer density AND smoking | 74 | Standard |
| 6 | Smoking OR tobacco AND density | 0 | Standard |

Search strategy and results: Web of Science

| **#** | **Searches** | **Results** | **Search Type** |
| --- | --- | --- | --- |
| 1 | Outlet density AND smoking | 383 | Standard |
| 2 | Retail density AND smoking | 197 | Standard |
| 3 | Smoking AND convenience store | 207 | Standard |
| 4 | Tobacco outlet density AND smoking | 268 | Standard |
| 5 | Tobacco retailer density AND smoking | 170 | Standard |
| 6 | Smoking OR tobacco AND density | 0 | Standard |

Search strategy and results: SocINDEX

| **#** | **Searches** | **Results** | **Search Type** |
| --- | --- | --- | --- |
| 1 | Outlet density AND smoking | 16 | Standard |
| 2 | Retail density AND smoking | 8 | Standard |
| 3 | Smoking AND convenience store | 13 | Standard |
| 4 | Tobacco outlet density AND smoking | 14 | Standard |
| 5 | Tobacco retailer density AND smoking | 6 | Standard |
| 6 | Smoking OR tobacco AND density | 0 | Standard |
