# Supplementary material for: Tobacco retailer density and smoking behaviour: how are exposure and outcome measures classified? A systematic review
Source: BMC Public Health. 2023 Oct 18;23:2038. doi: 10.1186/s12889-023-16914-y (PMC10585801; doi:10.1186/s12889-023-16914-y)
Supplement: Supplementary file 2 — Supplementary Material 2 [file 12889_2023_16914_MOESM2_ESM.docx]

| **Supplementary Table 2. Quality assessment of included studies** | | | | | |  |  |  |  |
| --- | --- | --- | --- | --- | --- | --- | --- | --- | --- |
| **Title** | **Author** | **Year** | **Data collection year** | **Country** | | **Study design** | **Sample size** | **Sample age** | **Quality assessment rating** |
| A comparison of individual versus community influences on youth smoking behaviours: a cross-sectional observational study | Adachi-Mejia, A. M.; Carlos, H. A.; Berke, E. M.; Tanski, S. E.; Sargent, J. D. | 2012 | 2007 | | USA | Cross-sectional | 3,646 | 13-18 years | Fair |
| A tale of two urbanicities: Adolescent alcohol and cigarette consumption in high and low-poverty urban neighborhoods | Davis, B; Grier, S. | 2015 | 2003-2005 | | USA | Cross-sectional | 10,000 | 12-17 years | Fair |
| Association Between Electronic Cigarette Marketing Near Schools and E-cigarette Use Among Youth | Giovenco, D.; Casseus, M; Duncan, D.; Coups, E.; Lewis, M.J; Delnevo, C. | 2016 | 2014 | | USA | Cross-sectional | 3,909 | 9-12 grade | Good |
| Banning tobacco sales and advertisements near educational institutions may reduce students' tobacco use risk: evidence from Mumbai, India | Mistry, R; Pednekar, M; Pimple, S; Gupta, P; McCarthy, W; Raute, L; Patel, M; Shastri, S. | 2015 | 2010 | | India | Cross-sectional | 1,533 | 8-10 grade | Good |
| Characteristics of tobacco retailers in New Zealand | Marsh, L; Doscher, C; Robertson, L. | 2013 | 2011-2012 | | New Zealand | Cross-sectional | NR | 15 years and over | Fair |
| Density and Proximity of Tobacco Outlets to Homes and Schools: Relations with Youth Cigarette Smoking | Lipperman-Kreda, S; Mair, C; Grube, J.; Friend, K.; Jackson, P; Watson, D | 2014 | 2009-2010 | | USA | Cross-sectional | 1,543 (2009) and 1,312 (2010) | 13-18 years | Good |
| Density of tobacco retail outlets near schools and smoking behaviour among secondary school students | Scully, M; McCarthy, M; Zacher, M; Warne, C; Wakefield, M; White, V. | 2013 | 2008 | | Australia | Cross-sectional | 2,044 | 12-17 years | Good |
| Density of Tobacco Retailers Near Schools: Effects on Tobacco Use Among Students | McCarthy, W; Mistry, R; Lu, Y; Patel, M; Zheng, H; Dietsch, B. | 2009 | 2003-2004 | | USA | Cross-sectional | 19,306 | Middle school and High school | Good |
| Does exposure to cigarette brands increase the likelihood of adolescent e-cigarette use? A cross-sectional study | Best, C; van der Sluijs, W.; Haseen, F.; Eadie, D.; Stead, M.; MacKintosh, A. M.; Pearce, J.; Tisch, C.; MacGregor, A.; Amos, A.; Miller, M.; Frank, J.; Haw, S. | 2016 | 2014 | | United Kingdom | Cross-sectional | 1,404 | Secondary 2 (mean age 14.0 years) and Secondary 4 (mean age 15.9 years) | Good |
| E-cigarette use among students and e-cigarette specialty retailer presence near schools | Bostean, G; Crespi, C.; Vorapharuek, Patsornkarn; McCarthy, William J. | 2016 | 2013-2014 | | USA | Cross-sectional | 67,701 | Grades 7, 9 and 11 | Good |
| Effects of neighbourhood socioeconomic status and convenience store concentration on individual level smoking | Chuang, Y.; Cubbin, C.; Ahn, D.; Winkleby, M. A. | 2005 | 1979-1990 | | USA | Cross-sectional (multiple) | 8,121 | 25–74 years | Fair |
| Exploration of the Link Between Tobacco Retailers in School Neighborhoods and Student Smoking | Adams, M; Jason, L.; Pokorny, S; Hunt, Y. | 2013 | 2002 | | USA | Cross-sectional | 10,662 | Grades 7-10 | Good |
| Is adolescent smoking related to the density and proximity of tobacco outlets and retail cigarette advertising near schools? | Henriksen, L; Feighery, E; Schleicher, N.; Cowling, David W.; Kline, Randolph S.; Fortmann, Stephen P. | 2008 | 2005-2006 | | USA | Cross-sectional | 24,875 | High school | Good |
| Is neighbourhood access to tobacco outlets related to smoking behaviour and tobacco-related health outcomes and hospital admissions? | Barnes, R; Foster, S; Pereira, G; Villanueva, K; Wood, L. | 2016 | 2003–2009 | | Australia | Cross-sectional | 12,270 | 18 years and over | Good |
| Local Tobacco Policy and Tobacco Outlet Density: Associations With Youth Smoking | Lipperman-Kreda, S; Grube, J; Friend, K. | 2012 | 2000* and 2009* | | USA | Cross-sectional | 1,491 | 13-16 years | Good |
| Retail food availability, obesity, and cigarette smoking in rural communities | Hosler, A. | 2009 | 2003 and 2000-2005 | | USA | Cross-sectional | 274 | NR | Fair |
| Retail tobacco outlet density and youth cigarette smoking: a propensity-modeling approach | Novak, S; Reardon, S; Raudenbush, S; Buka, S. | 2006 | 1995-1999 | | USA | Cross-sectional* | 2,116 | 11-23 years | Good |
| Small-area estimation and prioritizing communities for tobacco control efforts in Massachusetts | Li, W; Land, T; Zhang, Z; Keithly, L; Kelsey, J. | 2009 | 1999-2005 | | USA | Cross-sectional* | 55,467 | 18 years and over | Good |
| Smoking and binge-drinking among adolescents, Ontario, Canada: Does the school neighbourhood matter? | Larsen, K; To, T; Irving, H; Boak, A; Hamilton, H; Mann, R; Schwartz, R; Faulkner, G. | 2017 | 2013 | | Canada | Cross-sectional | 6,142 | Grades 9-12 | Good |
| The added value of accounting for activity space when examining the association between tobacco retailer availability and smoking among young adults | Shareck, M; Kestens, Y; Vallee, J; Datta, G; Frohlich, K. | 2016 | 2011-2012 | | Canada | Cross-sectional | 1,994 | 18-25 years | Good |
| The association between the density of retail tobacco outlets, individual smoking status, neighbourhood socioeconomic status and school locations in New South Wales, Australia | Marashi-Pour, S; Cretikos, M; Lyons, C; Rose, N; Jalaludin, B; Smith, J. | 2015 | 2008-2011 | | Australia | Cross-sectional | 29,375 | 16 years and over | Good |
| The density of tobacco retailers and its association with attitudes toward smoking, exposure to point-of-sale tobacco advertising, cigarette purchasing, and smoking among New York youth | Loomis, B; Kim, A; Busey, A; Farrelly, M; Willett, J; Juster, H. | 2012 | 2000-2008 | | USA | Cross-sectional | 70,427 | 9-17 years | Fair |
| The density of tobacco retailers in home and school environments and relationship with adolescent smoking behaviours in Scotland | Shortt, N. K; Tisch, C; Pearce, J; Richardson, E. A; Mitchell, R. | 2016 | 2010-2011 | | United Kingdom | Cross-sectional | 20,446 | Secondary 2 and Secondary 4 | Fair |
| The Moderating Role of Gender in the Relationship Between Tobacco Outlet Exposure and Tobacco Use Among African American Young Adults | Brown, Q; Milam, A.; Bowie, J; Ialongo, N; Gaskin, D; Furr-Holden, D. | 2016 | 2009 | | USA | Cross-sectional | 283 | 21-24 years | Good |
| Tobacco outlet density and converted versus native non-daily cigarette use in a national US sample | Kirchner, T; Anesetti-Rothermel, A; Bennett, M; Gao, H; Carlos, H; Scheuermann, T; Reitzel, L; Ahluwalia, J. | 2017 | 2012 | | USA | Cross-sectional | 2,376 | 25 years and over | Fair |
| Tobacco outlet density and smoking prevalence: Does racial concentration matter? | Reid, R; Peterson, N; Lowe, John; Hughey, J. | 2005 | 2000-2002 | | USA | Cross-sectional | NR | Adults* | Fair |
| Tobacco outlet density, cigarette smoking prevalence, and demographics at the county level of analysis | Peterson, N; Lowe, J; Reid, R. | 2005 | 2000-2002 | | USA | Cross-sectional | 3,662 | 18 years and over | Good |
| Tobacco outlet density near home and school: Associations with smoking and norms among US teens | Schleicher, N; Johnson, T; Fortmann, S; Henriksen, L. | 2016 | 2011-2012 | | USA | Cross-sectional | 2,771 | 13-16 years | Good |
| Tobacco Point-of-Purchase Marketing in School Neighbourhoods and School Smoking Prevalence: A Descriptive Study | Lovato, C; Hsu, H; Sabiston, C; Hadd, V; Nykiforuk, C. | 2007 | NR | | Canada | Cross-sectional | 22,318 | Grades 10-11 | Fair |
| Tobacco Retail Environments and Social Inequalities in Individual-Level Smoking and Cessation Among Scottish Adults | Pearce, J; Rind, E; Shortt, N; Tisch, C; Mitchell, R. | 2016 | 2008-2012 | | United Kingdom | Cross-sectional | 28,751 | 16 years and over | Good |
| Tobacco retail outlet density and risk of youth smoking in New Zealand | Marsh, L; Ajmal, A; McGee, R; Robertson, L; Cameron, C; Doscher, C. | 2016 | 2012 | | New Zealand | Cross-sectional | 27,238 | 14-15 years | Good |
| Tobacco Retail Outlet Density and Young Adult Tobacco Initiation | Cantrell, J; Pearson, J. L; Anesetti-Rothermel, A; Xiao, H; Kirchner, T. R; Vallone, D. | 2016 | 2012-2013 | | USA | Cross-sectional | 4,288 | 18-34 years | Good |
| Tobacco retailer density surrounding schools and cigarette access behaviors among underage smoking students | Leatherdale, S; Strath, J. | 2007 | 2000-2001 | | Canada | Cross-sectional | 20,297 | Grades 9-12 | Good |
| Tobacco retailer density surrounding schools and youth smoking behaviour: a multi-level analysis | Chan, W; Leatherdale, S. | 2011 | 2005-2006 | | Canada | Cross-sectional | 26,924 | Grades 9-12 | Good |
| Using student and school factors to differentiate adolescent current smokers from experimental smokers in Canada: A multilevel analysis | Kaai, S; Leatherdale, S; Manske, S; Brown, S. | 2013 | 2008-2009 | | Canada | Cross-sectional | 5,440 | Grades 9-12 | Good |
| Tobacco retailer density and smoking behavior in a rural Australian jurisdiction without a tobacco retailer licensing system | Baker, J; Masood, M; Rahman, M.A; Thornton, L; Begg, S. | 2021 | 2019 | | Australia | Cross-sectional | 8,981 | 18 years and over | Good |
| Are Waterpipe Café, Vape Shop, and Traditional Tobacco Retailer Locations Associated with Community Composition and Young Adult Tobacco Use in North Carolina and Virginia? | King, J; Wagoner, K; Suerken, C; Song, E; Reboussin, B; Spangler, J; Walker, S; Cornacchione Ross, J; Wolfson, M; Sutfin, E. | 2020 | 2017 | | USA | Cross-sectional | 1,099 | Young adults* | Fair |
| Tobacco outlet density, neighbourhood stressors and smoking prevalence in Toronto, Canada | Kirst, Maritt; Chaiton, Michael; O'Campo, Patricia | 2019 | 2009-2011 | | Canada | Cross-sectional | 2,412 | 25-64 years | Good |
| Characterizing the spatial relationship between smoking status and tobacco retail exposure: Implications for policy development and evaluation | Brooks, M; Siegel, S; Curriero, F. | 2021 | 2018-2019 | | USA | Cross-sectional | 10,117 | Adults* | Fair |
| The Influence of Tobacco Retailer Density and Poverty on Tobacco Use in a Densely Populated Urban Environment | Farley, S; Maroko Andrew, R; Suglia, S; Thorpe, L. | 2019 | 2011-2013 | | USA | Cross-sectional | NR | 18 years and over | Fair |
| Associations between disparities in tobacco retailer density and disparities in tobacco use | Glasser, A; Onnen, N; Craigmile, P; Schwartz, E; Roberts, M. | 2022 | 2017-2019 | | USA | Cross-sectional | 73,359 | Middle School, High School, 11-16 years, 18 years and over | Fair |
| County-level associations between tobacco retailer density and smoking prevalence in the USA, 2012 | Golden, S; Kuo, T; Kong, A; Baggett, C; Henriksen, L; Ribisl, K. | 2020 | 2012 | | USA | Cross-sectional | NR | Adults* | Good |
| The influence of tobacco retailers on adolescent smoking: prevention and policy implications | Gwon, S. H; Yan, G; Huang, G; Kulbok, PA. | 2018 | 2015 | | South Korea | Cross-sectional | 714 | 13-15 years | Good |
| Associations of County Tobacco Retailer Availability With U.S. Adult Smoking Behaviors, 2014-2015 | Kong, A; Gottfredson, N; Ribisl, K; Baggett, C; Delamater, P; Golden, S. | 2021 | 2014-2015 | | USA | Cross-sectional | 88,850 | Adults* | Good |
| Youth daily exposure to tobacco outlets and cigarette smoking behaviors: Does exposure within activity space matter? | Lipperman-Kreda, S; Finan, L; Kowitt, S; Grube, J; Abadi, M; Balassone, A; Kaner, E. | 2020 | 2017-2018 | | USA | Cross-sectional | 100 | 16-20 years | Good |
| Smoking and Tobacco Retail Density Among Neighborhoods in Delaware | Pearce, M; Zucker, R; Lee, C; Kaur, O; McIntire, R. | 2019 | 2013 | | USA | Cross-sectional | 5,052 | Adults* | Fair |
| Association between school-based tobacco retailer exposures and young adolescent cigarette, cigar and e-cigarette use | Trapl, E; Anesetti-Rothermel, A; Moore, S. P; Gittleman, H. | 2021 | 2016 | | USA | Cross-sectional | 3,778 | Grades 7-8 | Good |

^NR^ =Not Reported. *Not specified.
