# Supplementary material for: Tobacco retailer density and smoking behaviour: how are exposure and outcome measures classified? A systematic review
Source: BMC Public Health. 2023 Oct 18;23:2038. doi: 10.1186/s12889-023-16914-y (PMC10585801; doi:10.1186/s12889-023-16914-y)
Supplement: Supplementary file 3 — Supplementary Material 3 [file 12889_2023_16914_MOESM3_ESM.docx]

### Supplementary Table 3. Smoking behaviour descriptions re-categorised

| **Smoking behaviour(s) assessed** | **Definition of smoking behaviour terms (summarised)** | **Re-categorised smoking behaviour** | **Authors** |
| --- | --- | --- | --- |
| **Ever-tried and Past-month (n=5)** | | | |
| Lifetime use | One or more times in life | Ever-tried | *Bostean et al. (2016)*[52] |
| Current | One or more times in the past 30 days | Past-month |  |
| Ever use | Ever used | Ever-tried | *Giovenco et al. (2016)*[53] |
| Past month | Number of days used in past 30 days | Past-month |  |
| Lifetime/Ever use combined | Number of cigarettes smoked in lifetime | Ever-tried | Adams et al. (2013)[37] |
| Current | Number of days smoked in past 30 days | Past-month |  |
| Ever use | Ever tried once or twice | Ever-tried | Mistry et al. (2015)[54] |
| Current | Any smoking in the past 30 days | Past-month |  |
| Lifetime smoking | Ever smoked in lifetime | Ever-tried | Gwon et al. (2018)[55] |
| Current | Number of days smoked in the last month | Past-month |  |
| **Ever-tried only (n=1)** | | | |
| Experimental/Daily combined | Smoked at least a few puffs of a cigarette on 2 or more days in the past month | Ever-tried | Lovato et al. (2007)[56] |
| **Past-month and Current (n=1)** | | | |
| Past month | Smoked in the last four weeks | Past-month | Scully et al. (2013)[36] |
| Past week | Number of cigarettes smoked on each of the last seven days (if any) | Current |  |
| **Past-month only (n=17)** | | | |
| Past month | Number of days smoked in past 30 days | Past-month | Davis et al. (2015)[45] |
| Current | Any smoking in the past 30 days,  number of cigarettes smoked in the past 30 days, number of days smoked, number of cigarettes per day | Past-month | Henriksen et al. (2008)[57] |
| Daily^af^^ | Smoked every day or almost every day in past 30 days | Past-month | Chan et al. (2011)[35] |
| Occasional^a^ | Smoked some days or only 1 or 2 days in past 30 days | Past-month |  |
| Current: Daily and Occasional combined^a^ | Smoked ≥1 cigarette per day in past 30 days/ Smoked ≥1 cigarette in past 30 days | Past-month | Kaai et al. (2013)[58] |
| Experimental | Smoked in past 30 days | Past-month |  |
| Past month | Number of days smoked in past 30 days | Past-month | Lipperman-Kreda et al. (2014)[59] |
| Established^a^ | Smoked ≥1 cigarette in past 30 days, Average number of cigarettes on smoking days | Past-month | McCarthy et al. (2009)[60] |
| Experimental | Smoked ≥1 cigarette in past 30 days, Average number of cigarettes on smoking days | Past-month |  |
| Current^a^ | Any smoking in the past 30 days | Past-month | Marsh et al. (2016)[61] |
| Experimental | Any smoking in the past 30 days | Past-month |  |
| Converted non-daily | Previously smoked every  day but now smoke non-daily, Days smoked in past 30 days, Cigarettes per day on days smoked | Past-month | Kirchner et al. (2017)[62] |
| Native non-daily | Only smoke non-daily, Days smoked in past 30 days, Cigarettes per day on days smoked | Past-month |  |
| Current | Any smoking in the past 30 days | Past-month | Brown et al. (2016)[63] |
| Current^a^ | Smoking at least once a month | Past-month | Marsh et al. (2013)[64] |
| Occasional | Smoked ≥ once in past month but not everyday or almost everyday, no. of cigarettes usually smoked in a typical day of smoking, how often they smoke during the school day, how often they smoke on weekends | Past-month | Leatherdale et al. (2007)[65] |
| Regular | Smoked everyday or almost everyday in past month, no. of cigarettes usually smoked in a typical  day of smoking, how often they  smoke during the school day, how often they smoke on weekends | Past-month |  |
| Current | Smoked ≥1 day in past 30 days; Smoked on at least 20 days in the past 30 days; and number of cigarettes per day | Past-month | Loomis et al. (2012)[66] |
| Current | Any smoking in the past 30 days | Past-month | Novak et al. (2006)[67] |
| Ever tried/Current combined | Ever tried (including a puff) and current smokers who reported any smoking in past 30 days | Past-month | Schleicher et al. (2016)[5] |
| Ever tried | Number of cigarettes smoked in lifetime, number of days smoked in past 30 days | Past-month | Adachi-Mejia et al. (2012)[44] |
| Current | Smoking in the past 30 days | Past-month | Kirst et al. (2019)[68] |
| Current | Number of days smoked in past 30 days | Past-month | Trapl et al. (2021)[69] |
| **Current only (n=15)** | | | |
| Daily | Average number of cigarettes per day | Current | Chuang et al. (2005)[70] |
| Smoker | Daily or occasional smoking | Current | Barnes et al. (2016)[71] |
| Current | Currently smoke daily or occasionally | Current | Shareck et al. (2016)[72] |
| Current | Smoke every day or some days | Current | Hosler, A (2009)[29] |
| Current | Currently smoking | Current | Li et al. (2009)[73] |
| Smoking prevalence^a^ | Smoke every day or some days | Current | Reid et al. (2005)[74] |
| Smoking prevalence^a^ | Smoke every day or some days | Current | Peterson et al. (2005)[75] |
| Current | Daily or occasional smoking | Current | Marashi-Pour et al. (2015)[76] |
| Current | Smoke at all currently | Current | Pearce et al. (2016)[77] |
| Current | Current every day smoker, current some day smoker | Current | Brooks et al. (2021)[78] |
| Current smoking prevalence | Ever smoked >100 cigarettes, smoke every day or some days | Current | Farley et al. (2019)[79] |
| Smoking prevalence | Ever smoked >100 cigarettes in lifetime, smoke every day or some days | Current | Golden et al. (2020)[80] |
| Current | Smoke every day or some days | Current | Pearce et al. (2019)[81] |
| Current | Smoke every day or some days | Current | Kong et al. (2021)[28] |
| Daily | Any cigarette per day, number of cigarettes per day | Current | Lipperman-Kreda et al. (2020)[27] |
| **Past-year only (n=2)** | | | |
| Smoker | Smoked >1 cigarette in past 12 months | Past-year | Larsen et al. (2017)[82] |
| Past 6 month | Initiation of cigarette use in the past 6 months | Past-year | Cantrell et al. (2016)[3] |
| **Past-year and Past-month (n=1)** | | | |
| Past-month | Smoking in the past 30 days | Past-month | King et al. (2020)[83] |
| Past 6 months^b^ | Smoking in the past 6 months | Past-year |  |
| **Ever-tried, Past-year and Past-month (n=1)** | | | |
| Ever tried | Ever smoked whole cigarette | Ever-tried | Lipperman-Kreda et al. (2012)[84] |
| Past year | Smoking frequency in past 12 months | Past-year |  |
| Past month | Smoking frequency in past 30 days | Past-month |  |
| **Ever-tried and Current (n=3)** | | | |
| Ever tried^b^ | Tried, once or twice, sometimes use  (more than once a month) or use often (more than once a week) combined | Ever-tried | *Best et al. (2016)*[85] |
| Current | Current smoker | Current |  |
| Ever tried | Tried smoking during lifetime | Ever-tried | Shortt et al. (2016)[86] |
| Current | Smoke at all currently | Current |  |
| Experimental | Tried a few times but never smoked regularly | Ever-tried | Baker et al. (2021)[46] |
| Occasional | Occasional smoking | Current |  |
| Daily | Daily smoking | Current |  |
| **Ever-tried, Past-month and Current (n=1)** | | | |
| Ever-used^c^ | Any smoking in lifetime | Ever-tried | Glasser et al. (2022)[87] |
| Current^c^ | Any smoking in the past 30 days | Past-month |  |
| Current^d^ | Smoke every day or some days | Current |  |

^a^ Respondents reported smoking at least 100 cigarettes in lifetime, Italics refers to studies focusing on e-cigarette use only, ^b^ Refers to studies that focus on e-cigarette use and combustible cigarette use, ^c^ Smoking behaviour captured amongst youth only, ^d^ Smoking behaviour captured amongst adults only.
