# Supplementary material for: Tobacco retailer density and smoking behaviour: how are exposure and outcome measures classified? A systematic review
Source: BMC Public Health. 2023 Oct 18;23:2038. doi: 10.1186/s12889-023-16914-y (PMC10585801; doi:10.1186/s12889-023-16914-y)
Supplement: Supplementary file 4 — Supplementary Material 4 [file 12889_2023_16914_MOESM4_ESM.docx]

### Supplementary Table 4. Methods for measuring Tobacco Retailer Density

| **No. of retailers using circular buffers (n=14)** | | | |  |
| --- | --- | --- | --- | --- |
| **Geocoded location** | **Distance** |  | **Authors** | |
| School | 0.4km |  | *Bostean et al. (2016)* [52] | |
| School | 0.5km |  | Scully et al. (2013)[36] | |
| School | 0.8km |  | Davis et al. (2015)[45];  *Giovenco et al. (2016)^* [53];  Adams et al. (2013)[37];  Henriksen et al. (2008)[57]; Gwon et al. (2018)[55] | |
| Home | 0.8km |  | Brooks et al. (2021)[78] | |
| School | 1km |  | Lovato et al. (2007)[56];  Chan et al. (2011)[35];  Kaai et al. (2013)[58] | |
| Home, | 1.2km and 1.6km |  | Lipperman-Kreda et al. (2014)[59] | |
| School |  |  |  |  |
| School | 1.6km |  | McCarthy et al. (2009)[60] | |
| Home | 1.6km |  | Chuang et al. (2005)[70] | |
| **No. of retailers using polygons (n=9)** | |  |  | |
| **Geocoded location** | **Buffer size** |  | **Authors** | |
| School | 0.1km, 0.2km, 0.3km, 0.4km, 0.5km |  | Mistry et al. (2015)[54] | |
| Home | 0.4km |  | Brown et al. (2016)[63] | |
| Home, | 0.5km |  | Shareck et al. (2016)[72] | |
| Activity Space |  |  |  |  |
| School | 0.5km and 1km |  | Marsh et al. (2013)[64];  Marsh et al. (2016)[61] | |
| Home | 1.6km |  | Barnes et al. (2016)[71] | |
| School | 1.6km |  | Larsen et al. (2017)[82] | |
| School | Six-block radius |  | Leatherdale et al. (2007)[65] | |
| Activity Space | 0.1km |  | Lipperman-Kreda et al. (2020)[27] | |
| **No. of retailers per population (n=10)** | |  |  | |
| **Area** | **Per population** |  | **Authors** | |
| County | Per 1,000 youth |  | Loomis et al. (2012)[66] | |
| City | Sample of 2,116 young people from 178 Census tracts within 80 neighbourhood clusters |  | Novak et al. (2006)[67] | |
| City | Per 10,000 people |  | Lipperman-Kreda et al. (2012)[84] | |
| Census blocks | Per 10,000 people |  | Hosler, A (2009)[29] | |
| Census tract | Per 1,000 people |  | Glasser et al. (2022)[87] | |
| Census tract | Per 1,000 people |  | King et al. (2020)[83] | |
| County | Per 1,000 people |  | Kong et al. (2021)[28] | |
| Census tract | Per 1,000 people |  | Pearce et al. (2019)[81] | |
| County | Per 1,000 people |  | Golden et al. (2020)[80] | |
| County | Per 1,000 people |  | Brooks et al. (2021)[78] | |
| City | Per 1,000 people |  | Brooks et al. (2021)[78] | |
| **No. of retailers per km of roadway (n=5)** | | | | |
| **Area** | **Per km of roadway** |  | **Authors** | |
| Neighbourhood planning districts | Per 1.6km of roadway |  | Li et al. (2009)[73] | |
| Neighbourhood (census tract) | Per 10 kilometres of roadway |  | Cantrell et al. (2016)[3] | |
| County | Per 50 kilometres of roadway |  | Reid et al. (2005)^[74]^ | |
| County | Divided by kilometres of roadway |  | Peterson et al. (2005)[75] | |
| Home, School | No. of retailers within each roadway network buffer divided by the land area (square miles) |  | Schleicher et al. (2016)[5] | |
| **No. of retailers per population using KDE (n=4)** | | | | |
| **Area** | **Per population using KDE** |  | **Authors** | |
| Home | Individual measure of proximity-weighted retailer density within 800m radius of geocoded location for each postcode |  | *Best et al. (2016)^b^* [85] | |
| Home | No. of retailers per 1,000 people using adaptive bandwidth KDE |  | Adachi-Mejia et al. (2012)[44] | |
| School, Neighbourhood (postal area) | KDE per 1,000 people; median retailer density around schools |  | Marashi-Pour et al. (2015)[76] | |
| County | No. of retailers per 1,000 people using adaptive bandwidth KDE |  | Golden et al. (2020)[80] | |
| **No. of retailers per-km^2^ using KDE (n=4)** | |  |  | |
| **Area** | **Per km^2^ using KDE** |  | **Authors** | |
| Home, School | Proximity-weighted retailers per-km^2^ for each postcode using KDE |  | Shortt et al. (2016)[86] | |
| Neighbourhood (postcode) | Proximity-weighted retailers per-km^2^ for each postcode using KDE |  | Pearce et al. (2016)[77] | |
| School | Per 1.6 km^2^ using KDE |  | Trapl et al. (2021)[69] | |
| Neighbourhood | Proximity-weighted retailers per-1.6 km^2^ for each neighbourhood using KDE |  | Farley et al. (2019)[79] | |
| **No. of retailers per postcode using KDE (n=1)** | | | | |
| **Area** | **Per postcode using KDE** |  | **Authors** | |
| Neighbourhood | KDE to predict retailers and linked to participants’ residential ZIP code |  | Kirchner et al. (2017)[62] | |
| **No. of retailers per km^2^ (n=2)** | | | | |
| **Area** | **Per km^2^** |  | **Authors** | |
| Suburb | No. of retailers per km^2^ based on respondents’ suburb of residence |  | Baker et al. (2021)[46] | |
| Census tract | No. of retailers per km^2^ |  | Kirst et al. (2019)[68] | |

Italics refers to studies focusing on e-cigarette use only, ^b^Refers to studies that focus on e-cigarette use and combustible cigarette use.
